# Gas1 Regulates Patterning of the Murine and Human Dentitions through Sonic Hedgehog

**DOI:** 10.1177/00220345211049403

**Published:** 2021-11-19

**Authors:** M. Seppala, B. Thivichon-Prince, G.M. Xavier, N. Shaffie, I. Sangani, A.A. Birjandi, J. Rooney, J.N.S. Lau, R. Dhaliwal, O. Rossi, M.A. Riaz, D. Stonehouse-Smith, Y. Wang, S.N. Papageorgiou, L. Viriot, M.T. Cobourne

**Affiliations:** 1 Centre for Craniofacial & Regenerative Biology, Faculty of Dentistry, Oral & Craniofacial Sciences, King’s College London, London, UK; 2 Department of Orthodontics, Faculty of Dentistry, Oral & Craniofacial Sciences, King’s College London, London, UK; 3 Laboratoire de Biologie tissulaire et Ingénierie Thérapeutique (LBTI), UMR CNRS 5305/Université de Lyon 1, IBCP, Lyon, France; 4 Faculté d’Odontologie, Université de Lyon 1, Université de Lyon, Lyon, France; 5 Service d’Odontologie, Hospices Civils de Lyon, Lyon, France; 6 Clinic of Orthodontics and Pediatric Dentistry, Center of Dental Medicine, University of Zurich, Zurich, Switzerland

**Keywords:** supernumerary tooth, vestigial, molar cusp, molar size, molecular signaling, WNT

## Abstract

The mammalian dentition is a serially homogeneous structure that exhibits wide numerical and morphological variation among multiple different species. Patterning of the dentition is achieved through complex reiterative molecular signaling interactions that occur throughout the process of odontogenesis. The secreted signaling molecule Sonic hedgehog (Shh) plays a key role in this process, and the Shh coreceptor growth arrest-specific 1 (Gas1) is expressed in odontogenic mesenchyme and epithelium during multiple stages of tooth development. We show that mice engineered with *Gas1* loss-of-function mutation have variation in number, morphology, and size of teeth within their molar dentition. Specifically, supernumerary teeth with variable morphology are present mesial to the first molar with high penetrance, while molar teeth are characterized by the presence of both additional and absent cusps, combined with reduced dimensions and exacerbated by the presence of a supernumerary tooth. We demonstrate that the supernumerary tooth in *Gas1* mutant mice arises through proliferation and survival of vestigial tooth germs and that Gas1 function in cranial neural crest cells is essential for the regulation of tooth number, acting to restrict Wnt and downstream FGF signaling in odontogenic epithelium through facilitation of Shh signal transduction. Moreover, regulation of tooth number is independent of the additional Hedgehog coreceptors Cdon and Boc, which are also expressed in multiple regions of the developing tooth germ. Interestingly, further reduction of Hedgehog pathway activity in *Shh*^tm6Amc^ hypomorphic mice leads to fusion of the molar field and reduced prevalence of supernumerary teeth in a *Gas1* mutant background. Finally, we demonstrate defective coronal morphology and reduced coronal dimensions in the molar dentition of human subjects identified with pathogenic mutations in *GAS1* and *SHH/GAS1*, suggesting that regulation of Hedgehog signaling through GAS1 is also essential for normal patterning of the human dentition.

## Introduction

The mammalian dentition is a serially homologous structure defined by species-specific numerical and morphological variation achieved through reiterative molecular signaling during multiple stages of odontogenesis (Cobourne and Sharpe 2010; Jernvall and Thesleff 2012; Yu and Klein 2020). Specifically, signaling between oral ectoderm and cranial neural crest cell (CNCC)–derived (ecto)mesenchyme generates an ectodermal thickening that develops into a bud-stage tooth germ, which rapidly converts into cap and bell stages to establish and refine coronal shape (Jernvall and Thesleff 2000, 2012). The molecular interactions that drive odontogenesis are dominated by Wnt, bone morphogenetic protein, fibroblast growth factor (FGF), and Hedgehog signaling (Lan et al. 2014).

The modern human dental formula is reduced, having lost an incisor and 2 premolars during evolution (O’Leary et al. 2013), while the mouse dentition is highly reduced, being monophyodont and consisting of 1 incisor and 3 molars separated by an edentulous diastema (Prochazka et al. 2010). Detailed analysis of this diastema region has revealed paired vestigial tooth primordia appearing sequentially mesial to the first molar (M1) in both jaws from embryonic day (E)13.5 (Viriot et al. 2000; Peterková et al. 2002) (R1, R2; MS, R2 in maxilla and mandible, respectively) before disappearing at the bud stage through apoptosis (Peterková et al. 2003). R2 survival has been identified as the origin of supernumerary teeth present in the diastema of multiple mouse mutants (Kassai et al. 2005; Peterková et al. 2005; Klein et al. 2006; Ohazama et al. 2009; Ahn et al. 2010). A network of feedback inhibition restricts Wnt and downstream FGF activity in R2, preventing development beyond the bud stage and maintaining a reduced murine dental formula (Cobourne and Sharpe 2010; Lan et al. 2014). Specifically, canonical Lrp5/6-dependent Wnt signaling induces the secreted Wnt inhibitor Sostdc1 (Wise, Ectodin, or USAG-1) in dental mesenchyme to establish negative feedback in R2 (Ahn et al. 2010), while FGF signaling is restricted in epithelium and mesenchyme by Sprouty2/4 FGF inhibitor activity, respectively (Klein et al. 2006). Sonic hedgehog (Shh) is a target of Sostdc1-mediated Wnt signaling, contributing to Dkk1-mediated negative feedback acting to further inhibit R2 Wnt activity (Ahn et al. 2010). The precise role of Shh within this model is not fully understood, but a reaction-diffusion mechanism has been suggested, where temporospatial balance between Wnt and Hedgehog is responsible for tooth phenotype (Ahn et al. 2010; Cho et al. 2011).

Shh is a versatile signaling molecule mediating pathway activity through ligand binding to the Patched1 (Ptch1) receptor (Stone et al. 1996) facilitated by coreceptors including the GPI-anchored membrane protein Gas1 (growth arrest-specific 1) (Allen et al. 2007; Martinelli and Fan 2007) and immunoglobulin superfamily transmembrane proteins Cdon and Boc (Kang et al. 1997, 2002). These coreceptors can bind Shh (Okada et al. 2006; Tenzen et al. 2006; Martinelli and Fan 2007; McLellan et al. 2008) and Ptch1 (Bae et al. 2011; Izzi et al. 2011) and are collectively essential for vertebrate Hedgehog signaling (Allen et al. 2011).

Here we show that the *Gas1*^–/–^ molar dentition has abnormal coronal morphology and supernumerary teeth arising through R2 survival. *Gas1* function in CNCC inhibits Wnt signaling through facilitation of Shh to restrict tooth number. We also demonstrate defective coronal morphology in the molars of human subjects carrying missense mutations in *GAS1*, suggesting that regulation of SHH through GAS1 is also essential for patterning the human dentition.

## Materials and Methods

Experimental materials and methods are provided in the supplementary appendix material.

## Results

### Multiple Anomalies in the Dentition of Gas1^–/–^ Mice

We undertook *Gas1* expression analysis in the developing molar dentition within the context of Hedgehog activity (Appendix Fig. 1A–O). *Gas1* was initially expressed within mesenchyme peripheral to the molar tooth germ, progressively localizing in odontogenic mesenchyme adjacent to the regressing R2. By late cap stage, transcripts were also present in epithelium of the dental lamina and oral surface of the outer enamel epithelium. In addition, the mesenchymal expression was now localized to a region between the oral epithelium and oral surface of the tooth germ, extending to posterior regions of the buccal (vestibular) oral cavity. These expression domains were suggestive of a role in patterning and morphogenesis of the developing dentition.

**Figure 1.**
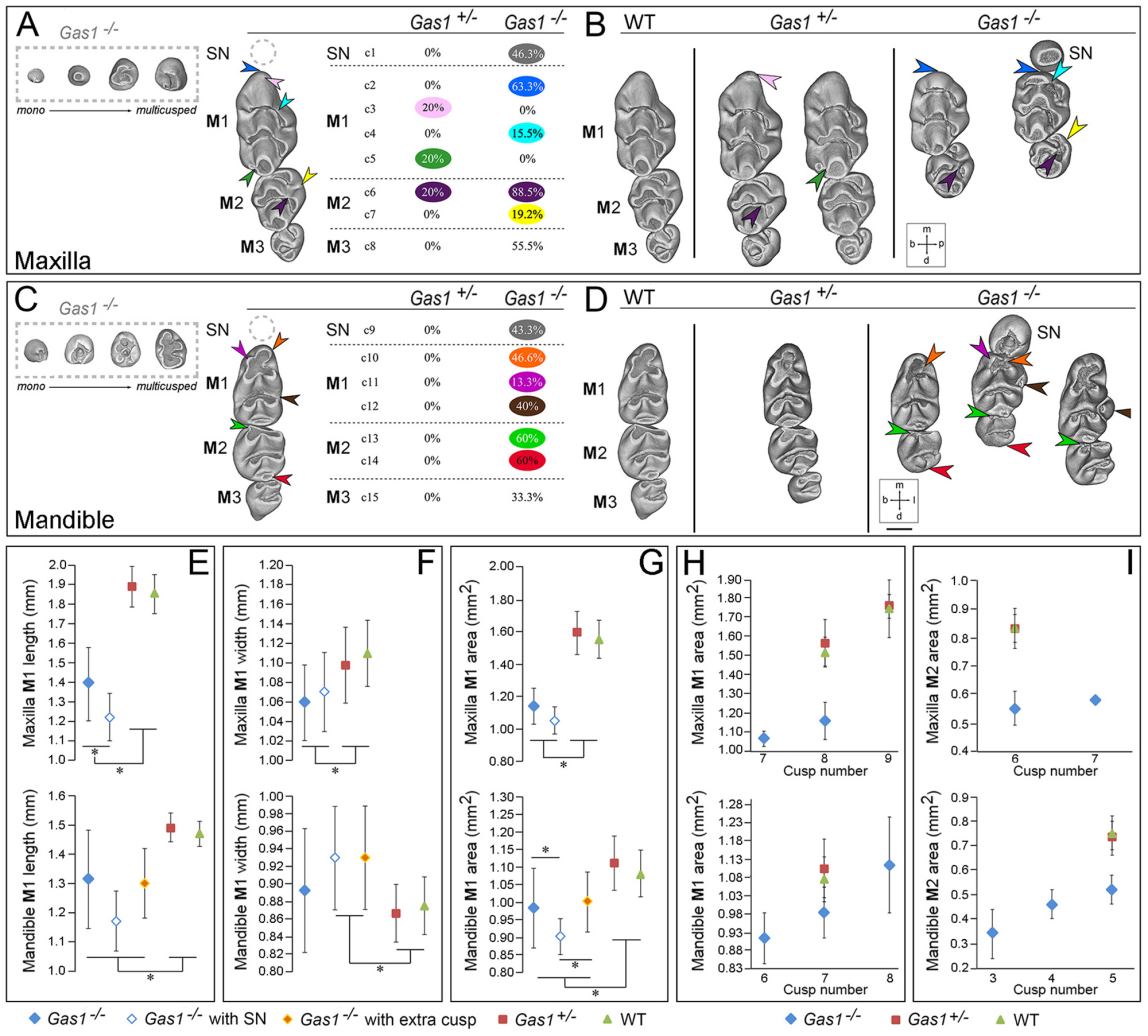
Dental character matrix of *Gas1* mutant mice. (**A**, **C**) The first column displays molar tooth rows with supernumerary tooth morphology variation shown within a gray hatched box and colored arrowheads localizing the most frequent defects in mutant backgrounds. The second column lists each character with the observed occurrence frequency in mutant mice. (A) Maxillary dental row (c1–8 are defects of the mutant maxillary molar dentition); c1, supernumerary tooth (50% of supernumerary teeth were associated with a missing M3 in the same row); c2, straight M1 mesial cusp with a vertical tilt of the mesial component (blue arrow); c3, M1 extra mesial cusp (pink arrow); c4, absence of M1 buccal cusp connection from the first chevron (turquoise arrow); c5, connection pinching to disconnection of the M1 third chevron cusps (dark green arrow); c6, disconnection of the M2 mesiolingual cusp from the first chevron (deep purple arrow); c7, abnormal connection between the 2 M2 lingual cusps (yellow arrow); c8, absence of M3. (C) Mandibular dental row (c9–15 are defects of the mutant mandibular molar dentition); c9 supernumerary tooth (37.5% of supernumerary teeth were associated with a missing M3 in the same row); c10, absence of the M1 first lingual cusp (orange arrow); c11, absence of the M1 first buccal cusp (light purple arrow); c12, presence of an extra M1 first lingual cusp (brown arrow); c13, absence of the M3 mesiolingual cingulum (light green arrow); c14, absence of the M2 distal-most cusp (red arrow); c15, absence of M3. Molar phenotype of wild-type (WT), *Gas1*^+/–^, and *Gas1*^–/–^ mice. (**B**, **D**) WT, *Gas1*^+/–^, and *Gas1*^–/–^ molar tooth rows are shown in the 3 columns (respectively from left to right). (B) For the maxillary molar dentition: straight M1 mesial cusp associated with a vertical tilt of the mesial component (blue arrow), M1 extra mesial cusp (pink arrow), absence of the M1 palatal cusp connection from the first chevron (turquoise arrow), connection pinching to disconnection of the M1 third chevron cusps (dark green arrow), disconnection of the M2 mesiopalatal cusp from the first chevron (deep purple arrow), and abnormal connection between the 2 M2 palatal cusps (yellow arrow). (D) For the mandibular molar dentition: absence of the M1 first lingual cusp (orange arrow), absence of the M1 first buccal cusp (light purple arrow), presence of an extra M1 first lingual cusp (brown arrow), absence of the M2 first chevron cusp (light green arrow), and absence of the M2 most distal step (red arrow). (E–G) Maxillary and mandibular molar dimensions in WT, *Gas1+/-* and *Gas1*^–/–^ mice. (E) M1 mesiodistal length (mm). (F) M1 buccal-lingual width (mm). (G) M1 coronal surface area (mm^2^). (H, I) Maxillary and mandibular M1 and M2 coronal surface area and cusp number. (H) M1. (I) M2. For (E) to (I), maxillary teeth are shown in the upper part of the panel and mandibular in the lower. Scale bar in D = 0.45 mm for (A) to (D). M1, M2, M3, first, second, third molar, respectively; SN, supernumerary tooth. b, buccal; d, distal; l, lingual (mandible); m, mesial; p, palatal (maxilla). Blue solid diamond, *Gas1*^–/–^; blue outline diamond, *Gas1*^–/–^ with supernumerary tooth; green triangle, WT; orange diamond, *Gas1*^–/–^ with extra cusp; red square, *Gas1*^+/–^. *Indicates significant difference (*P* < 0.05).

To investigate the role of *Gas1* during dental development, we compared arrangement and shape of molar tooth rows in maxilla and mandible of wild-type (WT) and *Gas1* mutant mice (Fig. 1A–D). In our sample, 80% of *Gas1*^–/–^ mice had a supernumerary tooth mesial to M1 (maxilla 46.3%; mandible 43.3%), which exhibited wide morphological variation (Fig. 1A, C; gray hatched box). However, 55.5% of mutants had absence of at least 1 M3, 55.5% and 33.3% were missing in the maxillary and mandibular molar tooth rows, respectively. In the maxilla, 50% of supernumerary teeth were associated with a missing M3 in the same row, while in the mandible, this was 37.5%.

All *Gas1*^–/–^ molar dentitions displayed anomalies in occlusal morphology, with less severe variation in heterozygotes. In the *Gas1*^–/–^ maxilla, the M1 mesiocentral cusp tilt was subvertical (63.3% overall, all M1 associated with a supernumerary) (Fig. 1A, B; blue arrows), and a palatal cusp connection within the first chevron was absent (15.5%) (Fig. 1A, B; turquoise arrows). In heterozygotes, 20% of maxillary M1 had an extra mesial cusp (Fig. 1A, B; pink arrows) as well as a connection anomaly between both cusps of the third chevron (Fig. 1A, B; dark green arrows). The maxillary M2 in both *Gas1*^–/–^ and heterozygous mice had a frequent disconnection of the mesiopalatal cusp from the first chevron (88.5% mutant; 20% heterozygote) (Fig. 1A, B; deep purple arrows) sometimes associated with an abnormal connection between the 2 palatal cusps (Fig. 1A, B, yellow arrows). In the mandible, heterozygous molars were essentially normal (Fig. 1C, D), but the *Gas1*^–/–^ M1 had a missing buccal cusp in 13%, as well as an extra lingual cusp in 40% (Fig. 1C, D; light purple and brown arrows, respectively), and lacked the mesiolingual cusp (46.6% overall, 92.3% associated with a supernumerary) (Fig. 1C, D; orange arrows) and, more rarely, the mesiobuccal cusp (13.3% overall, 7.7% associated with a supernumerary). In addition, 60% of *Gas1*^–/–^ mandibular M2 lacked the mesiobuccal cingulum and distal-most cusp (Fig. 1C, D; light green and red arrows, respectively).

Dimensional variations between M1 of WT and *Gas1*^–/–^ mice were also present. In both maxilla and mandible M1, length, width, and tooth surface area was reduced in mutants compared to heterozygote and WT, with M1 length further reduced in the presence of a supernumerary (Fig. 1E–G). M1 dimensions in *Gas1*^–/–^ were 25% and 9% smaller than WT in the maxilla and mandible, respectively, while M2 was 34% and 37% smaller. Interestingly, cusp number increased with tooth occlusal surface area regardless of genotype. However, comparing WT and *Gas1*^–/–^ teeth demonstrated that very different surface areas could contain the same number of cusps (Fig. 1H, I).

### Supernumerary Teeth in Gas1^–/–^ Mice Are a Product of the R2 Vestigial Tooth Bud

**Figure 2.**
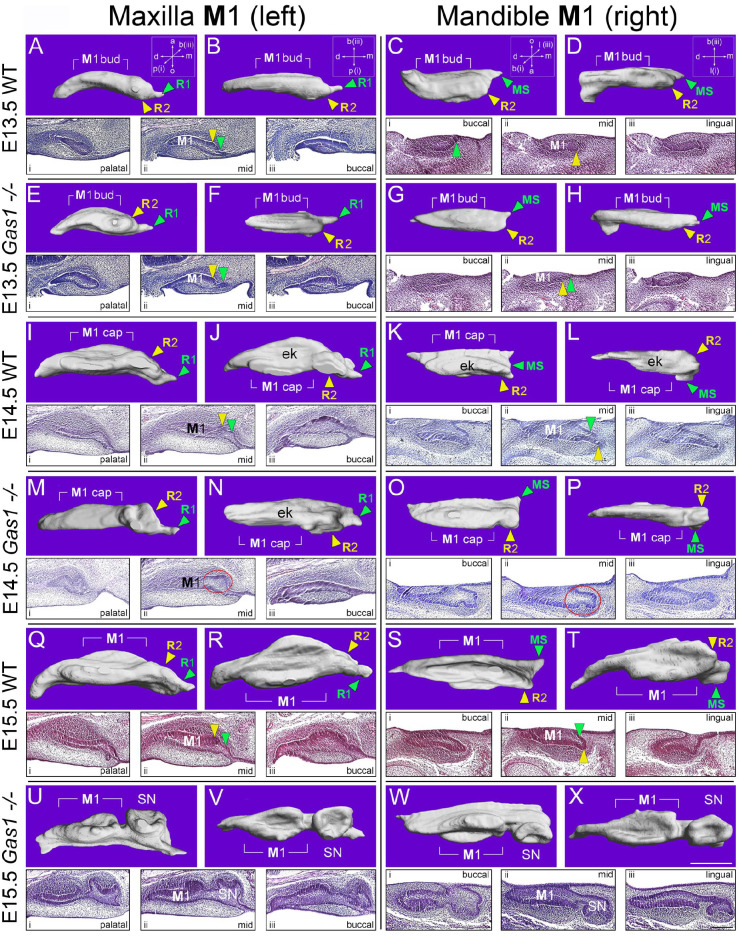
Supernumerary teeth in *Gas1*^–/–^ mice are a product of the R2 vestigial tooth germ. 3D reconstructions of the epithelial component and serial parasagittal histology of the left maxillary and right mandibular M1 in wild-type (WT) and *Gas1*^–/–^ mice. For the maxilla, (**A**, **E**, **I**, **M**, **Q**, **U**) show 3D reconstruction of M1 from the palatal aspect, while (**B**, **F**, **J**, **N**, **R**, **V**) are orientated from below. For the mandible, (**C**, **G**, **K**, **O**, **S**, **W**) show 3D reconstruction from the buccal aspect, while (**D**, **H**, **L**, **P**, **T**, **X**) are viewed from below. Serial histology is orientated from palatal through mid to buccal aspects (i, ii, iii; respectively) for the left maxillary M1 and from buccal through mid to lingual aspects (i, ii, iii; respectively) for the right mandibular M1. All images are orientated with mesial to the right. It can be seen that R1 and MS degenerate in the maxillary and mandibular M1 of WT and Gas1–/– mice. However, while R2 degenerates in the WT maxillary and mandibular M1, in the *Gas1*–/–, this vestigial tooth bud survives and goes on to form a supernumerary tooth in the maxilla and mandible. Scale bar in X = 150 µm for (A) to (X) and in (X) iii (lingual) = 100 µm for all histological sections. ek, primary enamel knot; M1, first molar; SN, supernumerary tooth. a, aboral; b, buccal; d, distal; m, mesial; o, oral; p, palatal. Green arrowhead, MS (maxilla) and R1 (mandible); red circle highlights developing R2 in the mutant tooth germ; yellow arrowhead, R2 (maxilla and mandible).

We next investigated developmental origins of supernumerary teeth in *Gas1*^–/–^ mice using 3-dimensional (3D) reconstruction (Fig. 2A–X). At E13.5, vestigial primordia were visible mesial to the bud-stage M1 with no obvious morphological differences between WT and mutant (Fig. 2A–D, E–H). At E14.5, R2 was still identifiable anterior to the cap-stage M1 in WT, although some incorporation into the M1 cap was evident in the mandible (Prochazka et al. 2010; Viriot et al. 2000) (Fig. 2I, J–L). In *Gas1*^–/–^ mice, R2 development continued toward an independent rudimentary cap stage in both arches, accompanied by delayed M1 cap formation (Fig. 2M–P). At E15.5, the diastema buds were no longer distinct entities in WT, particularly in the mandible, where R2 was now a component of the M1 cap (Fig. 2Q–T). However, cap-stage supernumerary tooth germs were present in *Gas1*^–/–^ embryos, anterior to a diminutive M1 cap-stage tooth germ in both arches (Fig. 2U–X). These findings suggested the developmental basis of *Gas1*^–/–^ supernumerary teeth was survival of R2, a finding confirmed by comparison of *Shh* expression in 3D M1 reconstructions (Appendix Fig. 2A–F) and analysis of proliferation and cell death. At E13.5, there was significantly more proliferation in the mutant R2 epithelium compared to WT, which continued in the epithelium and mesenchyme at E14.5. In addition, increased cell death was identified in the WT R2 epithelium compared to the mutant at E14.5 (Appendix Fig. 3A-J).

### Gas1 Regulates Tooth Number through the Shh Pathway

**Figure 3.**
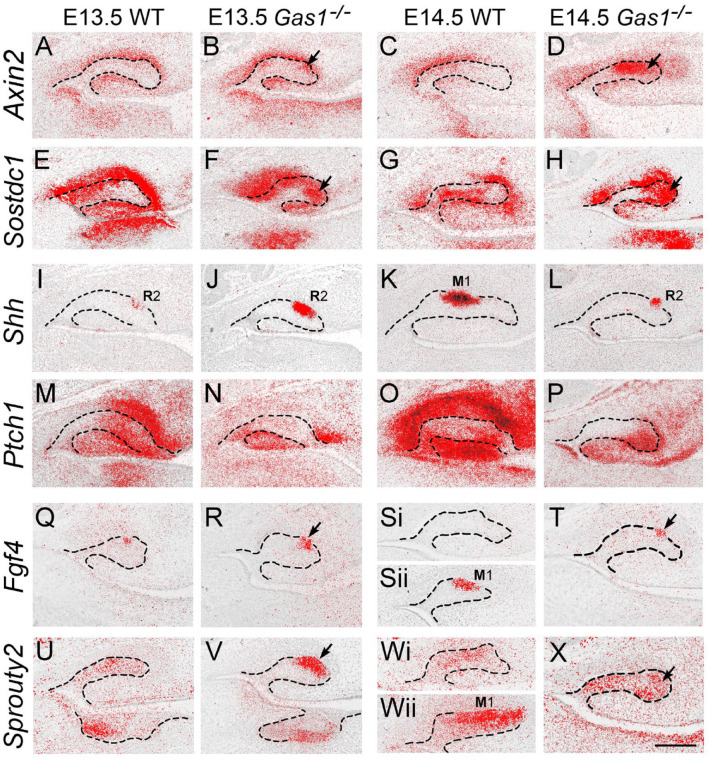
Increased WNT signaling is associated with reduced Sonic hedgehog (Shh) transduction and increased fibroblast growth factor (FGF) signaling in the developing R2 of Gas1–/– mice. ^35^S radiolabeled in situ hybridization on parasagittal sections through the developing maxillary molar tooth germs at E13.5 and E14.5. (**A–D**) *Axin2*. (**E–H**) *Sostdc1*. (**I–L**) *Shh*. (**M–P**) *Ptch1*. (**Q–T**) *Fgf4*. (**U–X**) *Sprouty2*. At E13.5 and E14.5, there was increased expression of *Axin2* and *Sostdc1* in the mutant R2 and proximate mesenchyme (A, B, C, D and E, F, G, H) (black arrows indicate increased expression in R2). In contrast, despite sustained transcription of *Shh* in the mutant R2 (I, J, K, L), *Ptch1* expression was reduced in the mutant (M, N, O, P). Consistent with a picture of increased Wnt signaling through reduced Shh inhibitory activity, the downstream Wnt targets *Fgf4* and *Sprouty2* were also increased in the mutant R2 compared to wild type (WT) at these stages, respectively (Q, R, S, T) and (U, V, W, X) (black arrows indicate increased expression in R2). Note a lack of *Fgf4* and *Sprouty2* expression in the WT R2 at E14.5 (Si and Wi, respectively) but expression of both genes in more lingual regions of the tooth germ in association with the M1 enamel knot (Sii and Wii, respectively). Hatched black line represents epithelial boundary. Black arrows indicate increased WNT signal transduction in R2. Scale bar in (X) = 100 µm for (A) to (X). Representative sections through WT and mutant maxillary tooth germs from *n* = 4 animals (*n* = 8 molars). M1, first molar enamel knot.

We next investigated Wnt, Hedgehog, and FGF signaling in WT and *Gas1*^–/–^ maxillary M1 between E13.5 and 14.5. In WT, *Axin2* was largely restricted to odontogenic mesenchyme, while *Sostdc1* was present in epithelium and mesenchyme (Fig. 3A, C, E, G). However, both these Wnt targets were ectopically expressed in the mutant R2 epithelium at both stages (Fig. 3B, D, F, H arrowed). Significantly, increased Wnt activity in the mutant R2 was associated with reduced Shh signal transduction. Despite sustained and increased *Shh* transcription in the mutant R2 compared to WT (Fig. 3I–L), *Ptch1* was reduced (Fig. 3M–P). At E13.5, increased *Fgf4* expression was also present in the mutant R2 compared to WT, while at E14.5, expression was lost in WT (although now present in M1) and retained in the mutant R2 (Fig. 3Q, R, S, T arrowed). A similar picture was seen in expression of the FGF target *Sprouty2*, which was also increased in the mutant R2 at E13.5 and increased at E14.5 in contrast to WT (Fig. 3U, V, W, X arrowed). An absence of Gas1 coreceptor function in mediating Shh signaling in odontogenic mesenchyme resulted in increased Wnt and FGF signaling in R2.

### Gas1 Regulates Tooth Number Independently through CNCC

**Figure 4.**
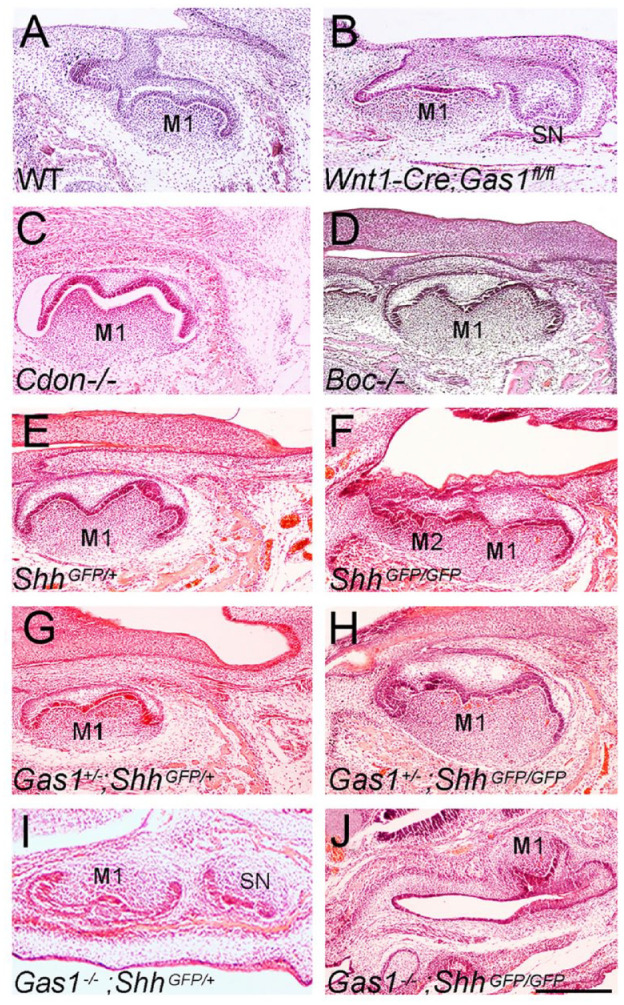
Molar phenotype of Hedgehog pathway mutant mice. (**A–D)** E16.5 (A, B) and E17.5 (C, D) mice lacking Hedgehog coreceptor function: (A) wild type (WT); (B) *Wnt1-Cre;Gas1*^fl/fl^ (*n* = 3); (C) *Cdon*^–/–^(*n* = 6); (D) *Boc*^–/–^ (*n* = 9). (**E–J**) E17.5 (E) *Shh*^GFP/+^; (F) *Shh*^GFP/GFP^ (*n* = 8); (G) *Gas1*^+/–^;*Shh*^GFP/+^ (*n* = 8); (H) *Gas1*^+/–^; *Shh*^GFP/GFP^ (*n* = 8); (I) *Gas1*^–/–^;*Shh*^GFP/+^ (*n* = 4); and (J) *Gas1*^–/–^; *Shh*^GFP/GFP^ (*n* = 1). Loss of a single *Gas1* allele does not affect molar phenotype in a *Shh*^GFP/+^ background or alter molar fusion in a *Shh*^GFP/GFP^ background, while *Gas1*^–/–^; *Shh*^GFP/+^ mice have supernumerary teeth with reduced penetrance. *Gas1*^–/–^;*Shh*^GFP/GFP^ embryos are early embryonic lethal, with a lack of mandibular molar development and only a poorly formed molar tooth identifiable in the anterior maxilla. Scale bar in J = 250 µm for (A) to (J).

Given the early expression of *Gas1* in odontogenic mesenchyme localizing around R2 (see Appendix Fig. 1K–M), we investigated whether Gas1 function was essential in this tissue for regulation of tooth number. Specifically, we analyzed *Wnt1-Cre;Gas1*^fl/fl^ conditional mutant mice, among which all exhibited supernumerary teeth mesial to M1 (83% maxilla; 67% mandible) (Fig. 4A, B), demonstrating that *Gas1* is essential in murine CNCC for regulation of tooth number.

Gas1 is known to interact with the Hedgehog coreceptors Cdon and Boc in different developmental contexts (Allen et al. 2011), and all 3 are expressed in the developing tooth (Appendix Fig. 4M-T). We investigated the effect of individual loss of coreceptor function on tooth development. Notably, at E18.5, there was no evidence of supernumerary tooth formation in either *Cdon*^–/–^ or *Boc*^–/–^ single mutants (Fig. 4C, D).

Given the independent role of Gas1 and reduced levels of Shh transduction observed in R2 of *Gas1* mutants (see Fig. 3M–P), we investigated the consequences of further reducing Hedgehog signal levels in this mutant background. *Gas1*^+/–^; *Shh*^+/–^ mice are normal, but loss of both *Shh* alleles in a *Gas1*^–/–^ background leads to gross craniofacial defects (Seppala et al. 2007). We therefore used the *Shh*^tm6Amc^ allele (*Shh*^GFP^), which encodes a bioactive Shh–green fluorescent protein (Shh::gfp)–tagged protein (Chamberlain et al. 2008). *Shh*^GFP/+^ mice are normal, but *Shh*^GFP/GFP^ mice are hypomorphic, and demonstrated M1–M2 fusion with complete penetrance but no supernumerary teeth (Fig. 4E, F). *Gas1*^+/–^;*Shh*^GFP/+^ mice had normal molar development while *Gas1*^+/–^;*Shh*^GFP/GFP^ maintained the molar fusion phenotype (Fig. 4G, H). However, *Gas1*^–/–^;*Shh*^GFP/+^ mice had supernumerary teeth with reduced penetrance (38%) in comparison to *Gas1*^–/–^, while *Gas1*^–/–^;*Shh*^GFP/GFP^ embryos were early embryonic lethal, with only 1 developing sufficiently to identify a lack of mandibular molars and only a poorly formed single molar in the anterior maxilla (Fig. 4I, J).

### Loss of Function in Hedgehog Signaling Affects Human Dental Development

**Figure 5.**
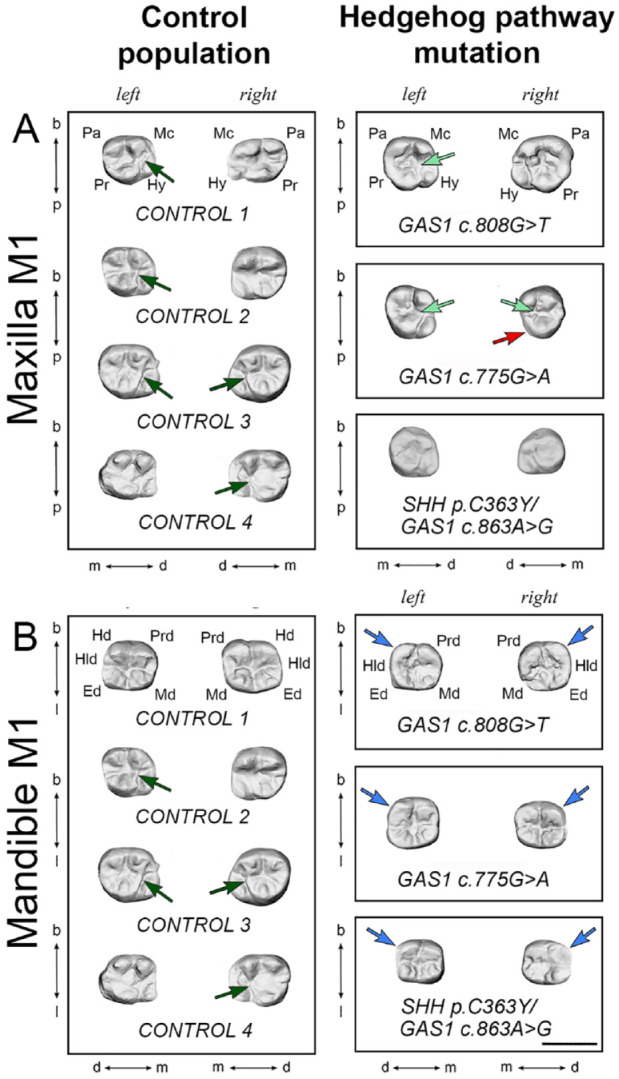
First permanent molar phenotype of human subjects with Hedgehog pathway mutation. (**A**) Maxillary and (**B**) mandibular left and right first permanent molars (M1) of representative control population (left panels) and human subjects with mutation in *GAS1* or *SHH/GAS1* (right panels). In the control population maxillary M1, a bridge separated the metacone and protocone in 5 of 8 teeth (dark green arrows), which was replaced by a groove in 3 of 6 subjects with mutations (light green arrows). In addition, the hypocone was absent in the right maxillary M1 in the subject with *GAS1* c.775G > A mutation (red arrow). In the mandible, the M1 hypoconulid was absent in the left and right M1 of all subjects with mutations (blue arrows). Scale bar in B = 1.0 cm for (A) and (B). Ed, entoconid; Hd, hypoconid; Hld, hypoconulid; Hy, hypocone; Mc, metacone; Md, metaconid; Pa, paracone; Pr, protocone; Prd, protoconid. B, buccal; d, distal; l, lingual (mandible); m, mesial; p, palatal (maxilla).

We next investigated the potential influence of GAS1-mediated Hedgehog signaling during human dental development by examining the permanent dentition of 3 subjects identified with pathogenic mutations at the *GAS1* (*n* = 2) or *SHH*/*GAS1* loci (*n* = 1) and features within the clinical spectrum of Holoprosencephaly (HPE), comparing them to population-matched controls (*n* = 4) (Ribeiro et al. 2010). The human M1 phenotype was characterized by significant mesiodistal shortening and an increased coronal width/length ratio (Appendix Fig. 5A–C). Moreover, there was absence of specific cusps and modification of interconnections between cusps when compared to population controls (Fig. 5A, B). Specifically, the mandibular M1 had absence of the distobuccal cusp (Hld, hypoconulid) in all subjects, while the maxillary M1 distopalatal cusp (Hy, hypocone) was reduced or absent in 1 subject (*GAS1* c.775G > A). The maxillary M1 in 2 of 3 subjects had a large marked groove separating the distobuccal (Mc, metacone) from the mesiopalatal cusp (Pr, protocone), which removed the enamel bridge usually present between these 2 cusps. There was also evidence of shoveling affecting the maxillary incisor crowns in 1 subject, taurodontism in 2 subjects, and generalized root shortening but no evidence of supernumerary teeth in the permanent dentition (data not shown).

## Discussion

We have identified morphological variation in the molar dentition of mice lacking function of the Hedgehog coreceptor Gas1 consistent with a model of altered Shh transduction. This included supernumerary tooth formation, altered cusp pattern, and size variation. Significantly, we have also have found cusp pattern and size defects in M1 of human subjects identified with pathogenic mutations in *GAS1* and *SHH*. These findings suggest some conservation of developmental pathways during patterning of the dentition.

### Supernumerary Tooth Formation in Gas1^–/–^ Mice

*Gas1*^–/–^ mice with the most severe craniofacial defects have supernumerary teeth in the molar dentition of both jaws with high penetrance (Seppala et al. 2007; Ohazama et al. 2009), while those surviving beyond birth have a prevalence of around 50% in both jaws. Among ERK-MAPK pathway mutants, *Sprouty2*^–/–^, *Sprouty4*^–/–^, and *Rsk2*^–/Y^ mice all have supernumerary teeth mesial to M1. *Sprouty2*^–/–^ have <5% in maxilla but >90% in mandible (Klein et al. 2006); *Sprouty4*^–/–^ and *Rsk2*^–/Y^ have 17% and 14%, respectively, in maxilla and 3% and 14%, respectively, in mandible (Klein et al. 2006; Marangoni et al. 2015). Among EDA pathway mutants, supernumerary teeth are present in 8% and 7% of molar rows in *Tabby*^/+^ and *Edar*^dl–j^ mice, respectively (Charles et al. 2009). Supernumerary teeth have also been reported in *Ectodin*^–/–^ and *Sostdc1*^–/–^ mutants with frequencies of 60% in both jaws reported for *Sostdc1*^–/–^ mice (Kassai et al. 2005). *Gas1*^–/–^ mice therefore represent mutants in which the frequencies of supernumerary tooth occurrence in the molar dentition are among the highest.

We have shown the developmental origin of *Gas1*^–/–^ supernumerary teeth to be R2 survival. Under normal circumstances, Wnt signaling establishes negative feedback in R2 through induction of Sostdc1 in dental mesenchyme (Ahn et al. 2010). Shh is a key target of Sostdc1-mediated Wnt signaling, contributing to this negative feedback through Wnt inhibition in R2 (Ahn et al. 2010). Wnt and Hedgehog activity is finely balanced in R2, which ultimately dictates developmental fate (Ahn et al. 2010; Cho et al. 2011). In the absence of Gas1, Shh transduction is reduced in M1 mesenchyme, and Wnt signaling in R2 elevates beyond the threshold required for survival. Indeed, maternal injection of the Shh-blocking antibody 5E1 or *Shh* downregulation in *PCS1-MRCS1*^Δ/Δ^ enhancer mutants also produces supernumerary teeth in the molar dentition (Cho et al. 2011; Seo et al. 2018; Kim et al. 2019). Interestingly, *Gas1*^–/–^ embryos demonstrate increased levels of *Sostdc1* activity in R2 epithelium compared to reporter activity seen in *Sostdc1* mutants (Ahn et al. 2010). In this model, relative levels of signaling within and around R2 are crucial and carefully regulated through multiple levels of negative feedback involving Wnt, FGF, and Hedgehog.

The importance of thresholds has been demonstrated through analysis of *Sostdc1* and *Shh*^GFP/Cre^ loss-of-function mice, in which individual heterozygotes are normal, but double heterozygotes have supernumerary teeth through increased Wnt and R2 survival (Ahn et al. 2010). In *Gas1*^–/–^ mice, Wnt signaling is increased because an absence of Gas1 reduces Shh signaling in odontogenic mesenchyme. However, the relationship with Shh signal transduction is complex because the transcriptional targets *Ptch1* and *Ptch2* negatively regulate signaling (Marigo et al. 1996; Chuang and McMahon 1999), while *Gas1*, *Cdon*, and *Boc* act positively but are negatively regulated by pathway activation (Tenzen et al. 2006; Martinelli and Fan 2007). Gas1 can also bind Ptch2 (Kim et al. 2020), but *Ptch2* expression is restricted to the enamel knot in the developing tooth germ (Motoyama et al. 1998). *Cdon* and *Boc* are individually redundant for molar tooth number regulation.

### Molecular Interactions Regulating Cusp Pattern

Shh has been shown to restrict cusp formation by regulating cusp spacing (Cho et al. 2011; Harjunmaa et al. 2012; Kim et al. 2019) and is also required for spatial patterning between individual molars (Cho et al. 2011). Embryos exposed to 5E1 have supernumerary cusps predominantly affecting maxillary M1 and mandibular M2 (Cho et al. 2011; Kim et al. 2019). *Gas1* heterozygotes demonstrated an additional maxillary M1 mesial cusp, while the mutant mandibular M1 had an additional lingual cusp. However, the mutant also had absence of some cusps, including the mandibular M1 mesiolingual and M2 first chevron buccal and distal-most cusps. This suggests a potential role for *Gas1* during evolutionary modulation of cusp morphology. The most severe molar pattern defects are associated with Shh inhibition from E14.5 (Cho et al. 2011; Kim et al. 2019). In *Gas1*^–/–^ mice, while there is a reduction in Shh signaling within the tooth germ, transduction is not completely lost, and therefore phenotypes might be expected to be more subtle.

The presence of a supernumerary tooth in *Gas1*^–/–^ mice directly affected the shape of the M1 mesial extremity in both jaws, with a corresponding size decrease in M1 through R2 tissue normally incorporated into this tooth contributing to an independent supernumerary. Shape diversity of the supernumerary was high in *Gas1*^–/–^ mice, ranging from tiny rounded and pointed teeth to larger, more complex crown forms that included alternately arranged cusps linked by a zigzag crest. This pattern variation has also been described in ERK-MAPK pathway mutants (Marangoni et al. 2015); therefore, no specific supernumerary tooth shape signature can be ascribed to *Gas1* mutants. However, a clear phenotypic signature in the molar dentition of *Gas1*^–/–^ mice was the presence of additional lingual cusps on the mandibular M1, which are never present in *Sprouty* and *Rsk* mutant mice (Charles et al. 2009; Marangoni et al. 2015) and are not known in the dentition of any living *Murinae*.

Are the supernumerary teeth seen in *Gas1*^–/–^ mice true premolars? The rodent molar dentition has a unity of shape, cuspal morphology, and arrangement that constitutes a morphological signature type. In rodents who have retained a premolar dentition (dormice, squirrels, porcupines), these teeth have a shape and cusp arrangement that very closely resemble that of molars. Direct comparison of a molar supernumerary from a mutant mouse is probably nonsensical because the mutant tooth has a shape and cusp arrangement that make it a supernumerary tooth of *Murinae*, and wild *Murinae* do not have premolars.

### GAS1 Function in Human Dental Development

The human subjects with mutation in *GAS1* or *GAS1/SHH* have an HPE craniofacial phenotype characterized by a flat face, maxillary and nasal hypoplasia, absent columella, and bilateral cleft lip/palate (Ribeiro et al. 2010). There was a general reduction in length of M1 in these subjects and absence of the mandibular M1 hypoconulid. This is a relatively rare phenotype in modern humans, representing around 0% to 10% of individuals with a maximum frequency of 20% in the populations of western Eurasia. The hypoconulid appeared relatively late in the evolution of mammals and was probably one of the last cusps to be established (Scott and Turner 1997). The maxillary hypocone was absent in 1 subject (*GAS1* c.775G > A). This is 1 of the 4 major cusps that compose the quadrangular permanent maxillary M1 and the last major cusp addition to the M1 crown during the evolution of primates. The absence of a M1 hypocone is therefore an extremely rare phenotype in humans, although this cusp may be reduced in some individuals (Scott and Turner 1997). Interestingly, reduction of the M1 hypocone and hypoconulid resembles the essential cusp pattern and shape of M2. These cusps are naturally reduced in M2 and may indicate a role for *GAS1* in mediating this process in humans.

## Author Contributions

M. Seppala, B. Thivichon-Prince, L. Viriot, M.T. Cobourne, contributed to conception, design, data acquisition, analysis, or interpretation, drafted and critically revised the manuscript; G.M. Xavier, contributed to conception, design, data acquisition, analysis, or interpretation, drafted the manuscript; N. Shaffie, I. Sangani, A.A. Birjandi, J. Rooney, J.N.S. Lau, R. Dhaliwal, O. Rossi, M.A. Riaz, D. Stonehouse-Smith, Y. Wang, S.N. Papageorgiou, contributed to data acquisition, analysis, or interpretation, drafted the manuscript. All authors gave final approval and agree to be accountable for all aspects of the work.

## Supplemental Material

sj-pdf-1-jdr-10.1177_00220345211049403 – Supplemental material for Gas1 Regulates Patterning of the Murine and Human Dentitions through Sonic Hedgehog Supplemental material, sj-pdf-1-jdr-10.1177_00220345211049403 for Gas1 Regulates Patterning of the Murine and Human Dentitions through Sonic Hedgehog by M. Seppala, B. Thivichon-Prince, G.M. Xavier, N. Shaffie, I. Sangani, A.A. Birjandi, J. Rooney, J.N.S. Lau, R. Dhaliwal, O. Rossi, M.A. Riaz, D. Stonehouse-Smith, Y. Wang, S.N. Papageorgiou, L. Viriot and M.T. Cobourne in Journal of Dental Research
