# Supplementary material for: Gas1 Regulates Patterning of the Murine and Human Dentitions through Sonic Hedgehog
Source: J Dent Res. 2021 Nov 19;101(4):473–82. doi: 10.1177/00220345211049403 (PMC8935464; doi:10.1177/00220345211049403)
Supplement: sj-pdf-1-jdr-10.1177_00220345211049403 – Supplemental material for Gas1 Regulates Patterning of the Murine and Human Dentitions through Sonic Hedgehog [file sj-pdf-1-jdr-10.1177_00220345211049403.pdf]

# Gas1 Regulates Patterning of the Murine and Human Dentitions through Sonic Hedgehog

Maisa Seppala, Beatrice Thivichon-Prince, Guilherme M. Xavier, Nina Shaffie, Indiya Sangani, Anahid A. Birjandi, Joshua Rooney, Jane N. S. Lau, Rajveer Dhaliwal, Ornella Rossi, Muhammed Adeel Riaz, Daniel Stonehouse-Smith; Yiran Wang; Spyridon N. Papageorgiou, Laurent Viriot, Martyn T. Cobourne

## Supplemental Appendix

## Materials and Methods

### *Mouse strains*

All animal procedures were performed under approved licence (PPL7007441) in accordance with the Animal (Scientific Procedures) Act, Her Majesty's Government, United Kingdom and local ethical approval at King's College London and conforms to ARRIVE guidelines. *Gas1*$^{+/-}$ (Lee et al. 2001); *Cdon*$^{+/-}$ (Okada et al. 2006); *Boc*$^{+/-}$ (Okada et al. 2006) and *Shh*$^{tm6Amc/+}$ (Chamberlain et al. 2008) mice were crossed to generate single and compound mutants; *Wnt1-Cre*$^{+/-}$ (Danielian et al. 1998) and *Gas1*$^{fl/+}$ (Jin et al. 2015) were crossed to generate conditional mutant mice, respectively in a mixed 129sv/C57BL/6 background.

For the analysis of erupted tooth morphology in post-natal (P) mice, *Gas1*$^{+/-}$ mice in a 129sv/CD1 background were used to generate single mutants (these mice present with a milder craniofacial phenotype and those without a cleft palate survive beyond birth). For histological and molecular analysis, *Gas1*$^{+/-}$ mice in a mixed 129sv/C57BL/6 background were used to generate single mutants (these mice have a more severe craniofacial phenotype, which includes cleft palate and do not survive beyond birth). All analysis of tooth number in these mice was conducted on embryonic material (E15.5 and beyond). All mice examined for supernumerary tooth prevalence (n=16) had evidence of at least two supernumerary teeth with 88% in the maxilla and 63% in the mandible. Timed-matings were set up such that noon of the day on which vaginal plugs were detected was considered as embryonic day (E) 0.5. In

a mixed 129sv/C57BL/6 background *Gas1;Shh*$^{GFP}$ homozygous mutants have severe developmental disruptions and essentially do not survive to E15.5.

### *Imaging of murine tooth rows*

The sample set for tooth row imaging consisted of 13 WT, 10 Gas1$^{+/-}$ and 15 Gas1$^{-/-}$ mice (a total of 152 dental rows or quadrants). The specimen age ranged from P2 weeks to 1 year, which meant that M3 analysis was only possible for mice >3 weeks of age (M3 is only mineralised from P20) (Cohn 1957). All non-mineralized tissues were removed to allow observation of the dental rows. Tooth rows were imaged using conventional X-ray microtomography with a Pheonix Nanotom (General Electrics) using the following parameters: 70 kV tensions, 100 mA intensity, 3000 images with time exposure of 500 ms and a voxel size of 3 µm. 3D renderings were performed using VG Studiomax software (Volume Graphics, Germany). The length, width and area of relevant tooth crowns were generated using ImageJ opensource software from occlusal-oriented pictures of the scanned volumes. More specifically, the crown surface area was calculated by drawing the outline of the molars. Since our measurements are based on the line of greatest contour of each molar, this allows us to disregard the varying effects of attrition depending on the age of the specimens. Kruskal Wallis test was used to verify the significance of differences in tooth size. A threshold p value of 0.05 was used to assess the significance of the observed differences.

### *Histological analysis and 3D reconstruction of embryonic tooth-germs*

For histological analysis, embryos were fixed in 4% paraformaldehyde (PFA) at 4°C, dehydrated through a graded ethanol series, embedded in paraffin wax, sectioned at 7 µm and stained with haematoxylin and eosin. Continuous para-sagittal sections of the developing maxillary and mandibular molar regions were imaged and photographed using an Axioskop 2plus microscope, AxioCam HRC camera and Axiovision 3.0 software (Zeiss, Germany). Images were imported into Adobe Photoshop CS6 (Adobe, USA) software, edited to highlight the epithelial component of the developing tooth-germs and saved. Images were imported into

DeltaViewer 2.1 3D Imaging software (freeware) and computationally stacked and aligned using the boundary of the oral epithelium and dental lamina as vertical and horizontal plane reference lines. In addition, continuous cross-referencing against the original histology was carried out throughout the process. The software reconstructed these images to produce a three-dimensional image of the developing molar tooth-germs whilst excluding the background. The reconstructed surface was then smoothened and saved as a QuickTime 7.7.5 (Apple Corp, USA) movie file. Static images were also taken from different views for each developing tooth (top, oral, anterior, buccal and palatal views) and finally montaged in Adobe Photoshop CS6 to facilitate ease of comparison between control and mutant molars. For *Shh* gene expression superimposition, continuous light-field images of $^{35}$S radioactive *in situ* hybridisation were taken and included with the relevant histology. Images were imported into Fiji Life-Line version 2015 software, stacked and manually aligned. Those regions corresponding to *Shh* expression were coloured red whilst grey was used to outline the tooth-germ. A total of n=5 *Gas1*$^{-/-}$ mutant embryos at E13.5 (n=1), E14.5 (n=3) and E15.5 (n=1) and stage-matched WT controls were collected. Each embryo allowed 3D analysis of n=4 quadrants, equating to a total of (n=20) quadrants for 3D analysis.

### ***In situ* hybridization**

Section *in situ* hybridization using $^{35}$S-UTP riboprobes was carried out as previously described (Gaete et al. 2015; Wilkinson 1992). Light and dark-field images of sections were photographed using a Zeiss Axioscop 2plus microscope and merged in Adobe Photoshop 2021. Sections are representative images from hybridization on continuous 7 µm sections through each tooth germ (a minimum of n=3 for each gene). cDNA was obtained from the following: Andrew McMahon, *Shh*; Matthew Scott, *Ptch1*; Chen-ming Fan, *Gas1*; Robert Krauss, *Cdon* and *Boc*; Atsushi Ohazama, *Fgf4*, *Sostdc1* and *Axin2*.

### ***Proliferation and TUNEL assays***

Bromodeoxyuridine (BrdU) labeling for cell proliferation was carried out on histological sections using a Zymed BrdU Labeling and Detection Kit (Invitrogen) according to the manufacturer's instructions. Mouse embryos were labelled with BrdU via intra-peritoneal injection into pregnant females (5mg/100g body weight) 2 hours prior to sacrifice. BrdU labelling in epithelium and mesenchyme at E13.5 and E14.5 was quantified manually by counting the proportion of BrdU-labelled cells within an area 100 $\mu m^2$ in 5 consecutive 7 μm para-sagittal sections through the R2 region of maxillary tooth-germs (n=4 WT and mutant animals, respectively).

Immunohistochemical detection of apoptotic cell death was carried out on histological sections using Terminal deoxynucleotidyl transferase-mediated deoxyUridine triphosphate Nick End Labeling (TUNEL). TUNEL was carried out using an APOPTag® Plus Fluorescein In Situ Apoptosis Detection Kit (Chemicon International) according to the manufacturer's instructions. TUNEL staining in the epithelium at E14.5 was quantified manually by counting the number of TUNEL-labelled cells within an area of 100 $\mu m^2$ in 5 consecutive 7 μm para-sagittal sections through the R2 region of maxillary tooth-germs (n=4 WT and mutant animals, respectively). All P-values were calculated using a Student's two-tailed t-test in Microsoft Excel.

***Analysis of human subjects***

The analysis of human subjects was previously approved by the Institutional Review Board of the Hospital de Reabilitaeao de Anomalias Craniofaciais, Bauru, Brazil (Ribeiro et al. 2010). Written informed consent was obtained from all parents and individuals included in the study. The human observational study conforms to STROBE guidelines. Human subjects with *GAS1* DNA sequence changes were identified from a sample of 54 individuals exhibiting features within the clinical spectrum of holoprosencephaly and a population-matched control sample with no history of familial structural anomalies of the central nervous system. Molecular analysis of genomic DNA identified the following variation in affected individuals: [Subject 1] was a female with missense mutation c.808G>T in *GAS1* (and her father had a Leu218Pro

mutation in *SHH*); [Subject 2] was a female with variation c.775G>A in *GAS1*; [Subject 3]: was a male with missense mutation p.C363Y in *SHH* and c.863A>G missense mutation in *GAS1*. The sequence analysis has been previously published with all identified *GAS1* variants considered to be damaging (Ribeiro et al. 2010). Dental panoramic radiographs and study casts were taken as part of routine dental care for these patients. Human maxillary and mandibular dental arches were imaged using 3Shape TRIOS scanners (3Shape, Denmark) from stone casts poured from maxillary and mandibular alginate impressions (n=3 per group). The length and width of M1 was measured using ImageJ software from occlusal-oriented pictures. Kruskal Wallis test was used to verify the significance of differences in tooth size. A threshold p value of 0.05 was used to assess the significance of the observed differences. The study protocol was not registered.

**Access to research materials**

Access requests for research materials used in this investigation should be addressed to MTC.

**References**

Chamberlain CE, Jeong J, Guo C, Allen BL, McMahon AP. 2008. Notochord-derived shh concentrates in close association with the apically positioned basal body in neural target cells and forms a dynamic gradient during neural patterning. Development. 135(6):1097-1106.

Cohn SA. 1957. Development of the molar teeth of the albino mouse. Am J Anat. 101:295-320.

Danielian PS, Muccino D, Rowitch DH, Michael SK, McMahon AP. 1998. Modification of gene activity in mouse embryos in utero by a tamoxifen-inducible form of cre recombinase. Curr Biol. 8(24):1323-1326.

Gaete M, Fons JM, Popa EM, Chatzeli L, Tucker AS. 2015. Epithelial topography for repetitive tooth formation. Biology open. 4(12):1625-1634.

Jin S, Martinelli DC, Zheng X, Tessier-Lavigne M, Fan CM. 2015. Gas1 is a receptor for sonic hedgehog to repel enteric axons. Proc Natl Acad Sci U S A. 112(1):E73-80.

Lee CS, May NR, Fan CM. 2001. Transdifferentiation of the ventral retinal pigmented epithelium to neural retina in the growth arrest specific gene 1 mutant. Dev Biol. 236(1):17-29.

Okada A, Charron F, Morin S, Shin DS, Wong K, Fabre PJ, Tessier-Lavigne M, McConnell SK. 2006. Boc is a receptor for sonic hedgehog in the guidance of commissural axons. Nature. 444(7117):369-373.

Ribeiro LA, Quiezi RG, Nascimento A, Bertolacini CP, Richieri-Costa A. 2010. Holoprosencephaly and holoprosencephaly-like phenotype and gas1 DNA sequence changes: Report of four brazilian patients. Am J Med Genet A. 152A(7):1688-1694.

Wilkinson DG. 1992. Whole mount in situ hybridisation of vertebrate embryos. In: Wilkinson DG, editor. In situ hybridisation:A practical approach. Oxford: Oxford University Press. p. 75-83.

**Supplementary Appendix Figures**

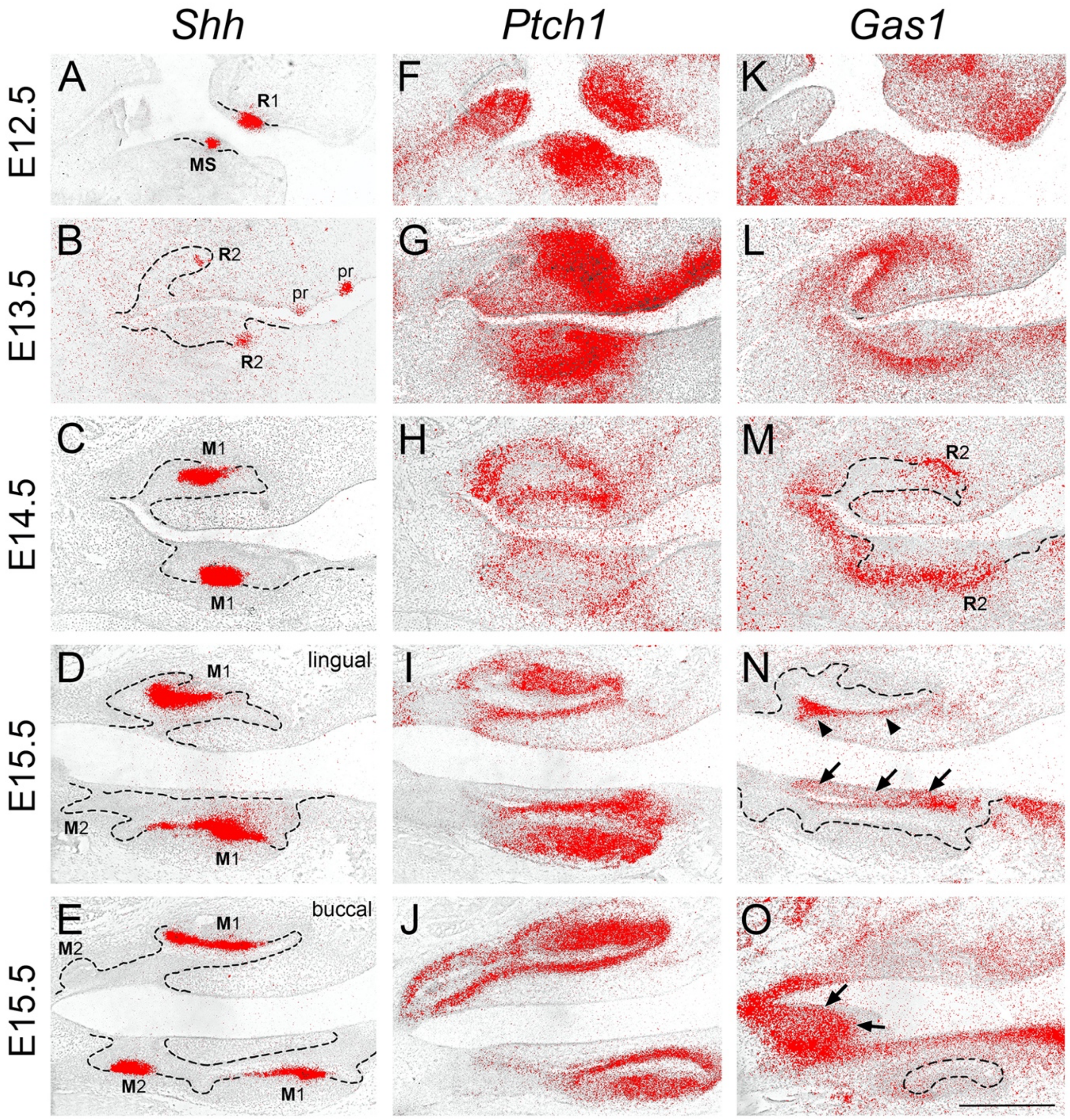


**Appendix Figure 1 Shh pathway expression in the developing molar tooth-germ.** $^{35}$S radio-labelled *in situ* hybridization on para-sagittal sections through the developing maxillary and mandibular molar tooth-germs from E12.5 to E15.5. (**A-E**) *Shh*; (**F-J**) *Ptch1*; (**K-O**) *Gas1*. Arrowheads in (**N**) indicate *Gas1* expression in the dental lamina and outer dental epithelium of the tooth-germ; arrows in (**N**, **O**) indicate *Gas1* expression in mesenchymal domains extending above (**N**) (lingually) and posterior (**O**) (buccally) to the molar tooth-germs. Hatched black line represents epithelial boundary. pr=palatine rugae. Scale bar in O=200 µm for A-O.

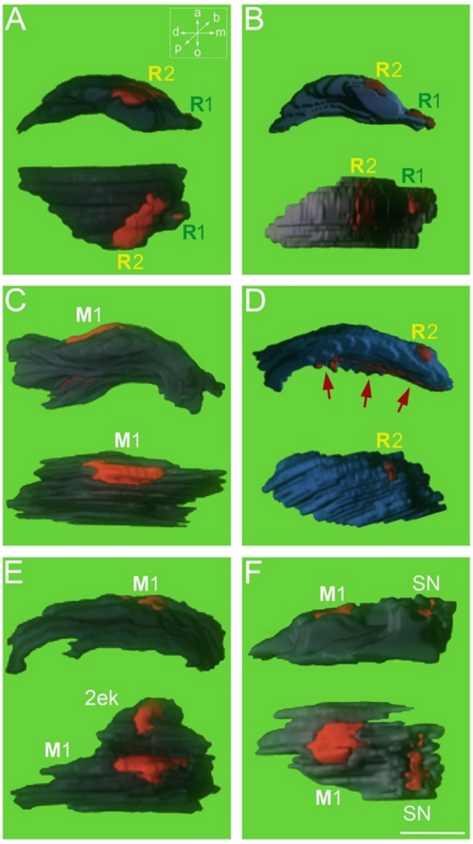


**Appendix Figure 2** ***Shh* expression during development of the maxillary molar tooth-germ.** 3D reconstructions of *Shh* expression (red) in the epithelial component (dark blue/grey) of maxillary molar tooth-germs derived from $^{35}$S radio-labelled *in situ* hybridization on para-sagittal sections. WT (left panels) and *Gas1*$^{-/-}$ (right panels). (**A**, **B**) E13.5; (**C**, **D**) E14.5; (**E**, **F**) E15.5 (palatal view above, aboral view below). In WT at E13.5, there was restricted *Shh* in R1 and a larger domain of in R2, whilst in the mutant R1 *Shh* was more prominent and a clear R2 domain was also present. At E14.5, the R2 domain had been lost in WT, whilst a large M1 domain was present in the enamel knot of the cap-stage tooth-germ. In the mutant at E14.5, there was evidence of prolonged R2 survival through continued *Shh* expression and delayed M1 development marked by an absence of *Shh* in the future enamel knot region. In addition, there was a small region of ectopic *Shh* expression spanning the oral side of the epithelial cap (Fig. 4D, red arrows). At E15.5, in WT the M1 domain began to regress whilst *Shh* expression appeared in secondary enamel knots; in the mutant, there was M1-associated *Shh* expression noted for the first time in the primary enamel knot and sustained expression in R2, which was now established as a cap stage supernumerary tooth-germ. M1=first molar; SN=supernumerary tooth; 2ek=secondary enamel knot. a=aboral; o=oral; m=mesial; d=distal; p=palatal; b=buccal. Scale bar in F=250 µm for A-F.

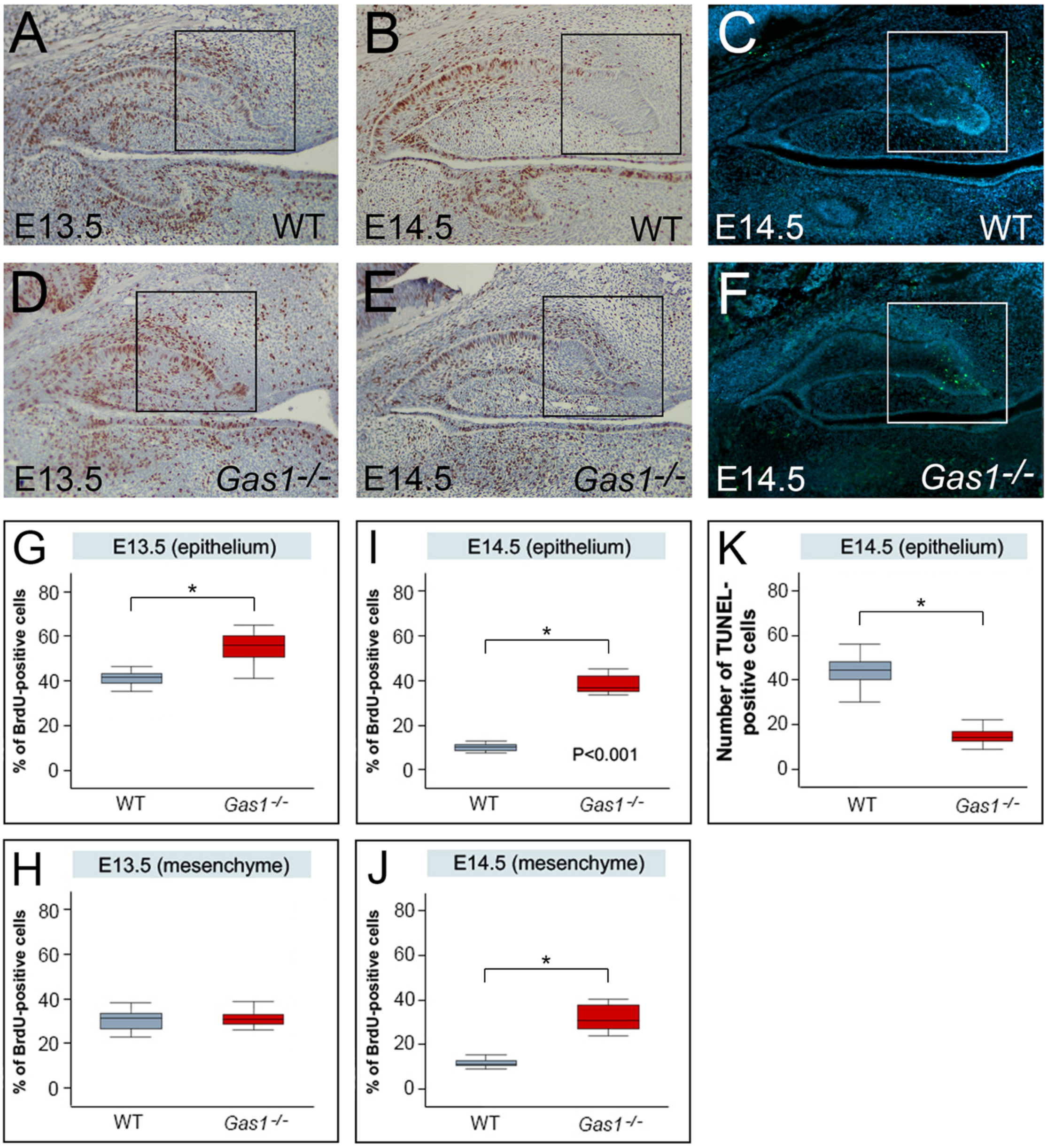


**Appendix Figure 3 Proliferation and apoptosis in the developing maxillary molar tooth-germ of WT and *Gas1*$^{-/-}$ mice.** (**A**, **B**; **D**, **E**) BrdU and (**C**, **F**) TUNEL staining on para-sagittal sections through M1 at E13.5 (**A**, **D**) and E14.5. (**B**, **E**; **C**, **F**). **Quantification of BrdU- and TUNEL-positive cells in R2. (G-J)** % BrdU-positive cells in the epithelium and mesenchyme of R2 at E13.5 and E14.5, respectively. (**K**) Number of TUNEL-positive cells in the epithelium of R2 at E14.5. There was a significantly increased % of BrdU-positive cells in R2 epithelium at E13.5 and both the epithelium and mesenchyme of R2 at E14.5 in the *Gas1*$^{-/-}$ compared to WT; whilst the number of TUNEL-positive cells was significantly increased in the WT R2 epithelium at E14.5 compared to the mutant. * P<0.001. Box in A-F=100 μm$^2$.

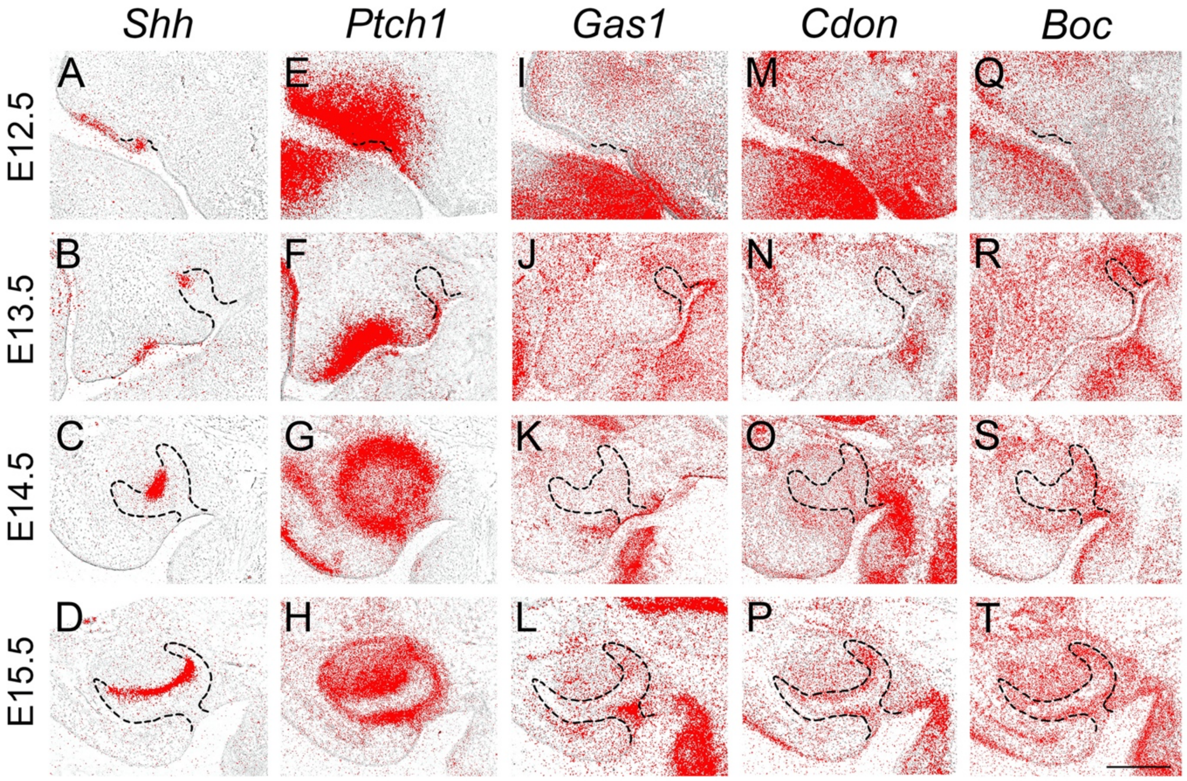


**Appendix Figure 4 Shh receptor expression in the developing molar tooth-germ.** Radioactive $^{35}S$ *in situ* hybridization on frontal sections through the developing maxillary M1 from E12.5 to 15.5. (**A-D**) *Shh*; (**E-H**) *Ptch1*; (**I-L**) *Gas1*; (**M-P**) *Cdon*; (**Q-T**) *Boc*. Scale bar in T=250 μm for A-T.

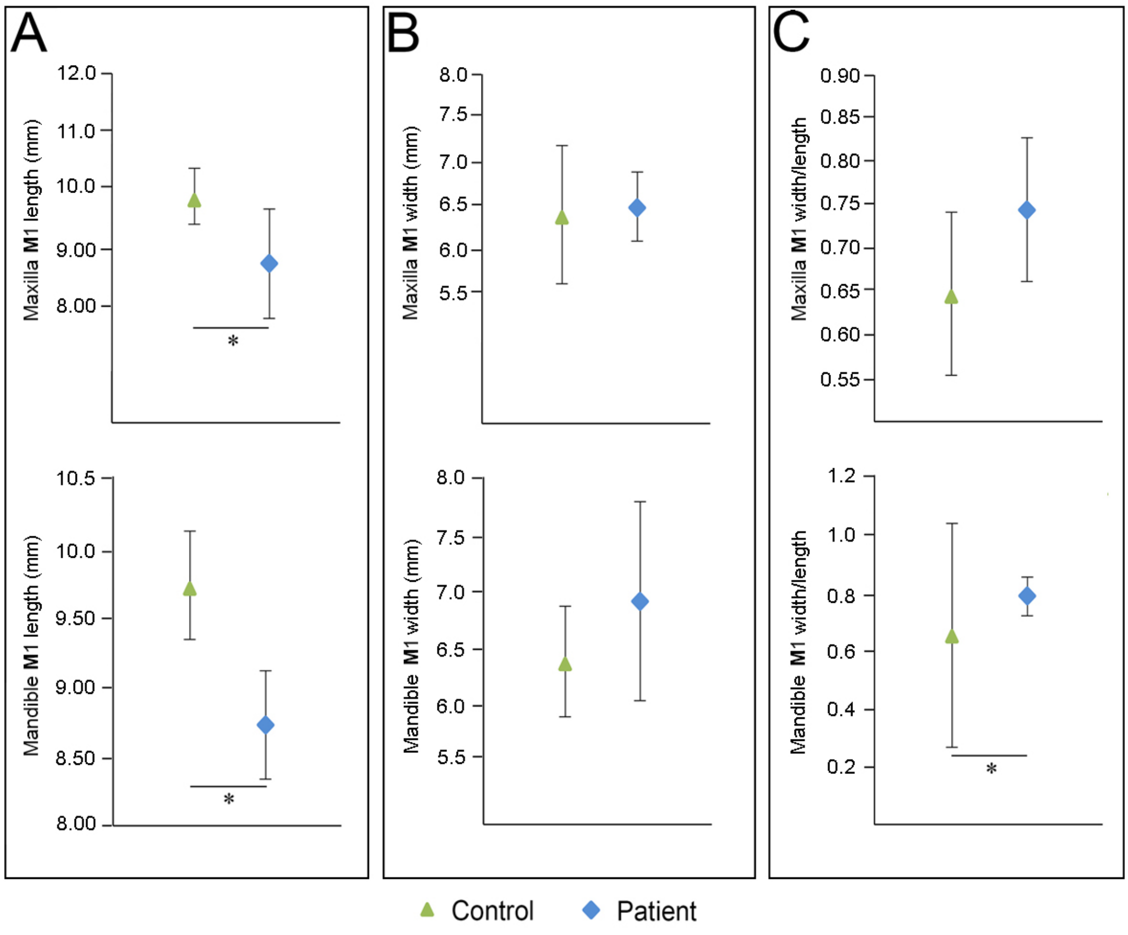


**Appendix Figure 5 Maxillary and mandibular M1 dental size in human control subjects and patients with *GAS1* mutation.** (**A**) M1 mesio-distal length (mm); (**B**) M1 buccal-lingual width (mm); (**C**) M1 width/length ratio. Maxillary M1 (upper panels) and mandibular M1 (lower panels). M1=first molar; green triangle=control subjects; blue solid diamond=patients with *GAS1/SHH* missense mutation. * indicates significant difference ($p<0.05$).
